# The retrocalcarine sulcus maps different retinotopic representations in macaques and humans

**DOI:** 10.1007/s00429-021-02427-0

**Published:** 2021-12-17

**Authors:** Michael J. Arcaro, Margaret S. Livingstone, Kendrick N. Kay, Kevin S. Weiner

**Affiliations:** 1 Department of Psychology, University of Pennsylvania, Philadelphia, PA 19146 USA; 2 Department of Neurobiology, Harvard Medical School, Boston, MA 02115 USA; 3 Center for Magnetic Resonance Research (CMRR), Department of Radiology, University of Minnesota, Minneapolis, MN 55455 USA; 4 Department of Psychology, University of California, Berkeley, Berkeley, CA 94720 USA; 5 Helen Wills Neuroscience Institute, University of California, Berkeley, Berkeley, CA 94720 USA

**Keywords:** Vision, Comparative neuroanatomy, Striate cortex, Calcarine sulcus, Human, Macaque

## Abstract

Primate cerebral cortex is highly convoluted with much of the cortical surface buried in sulcal folds. The origins of cortical folding and its functional relevance have been a major focus of systems and cognitive neuroscience, especially when considering stereotyped patterns of cortical folding that are shared across individuals within a primate species and across multiple species. However, foundational questions regarding organizing principles shared across species remain unanswered. Taking a cross-species comparative approach with a careful consideration of historical observations, we investigate cortical folding relative to primary visual cortex (area V1). We identify two macroanatomical structures—the retrocalcarine and external calcarine sulci—in 24 humans and 6 macaque monkeys. We show that within species, these sulci are identifiable in all individuals, fall on a similar part of the V1 retinotopic map, and thus, serve as anatomical landmarks predictive of functional organization. Yet, across species, the underlying eccentricity representations corresponding to these macroanatomical structures differ strikingly across humans and macaques. Thus, the correspondence between retinotopic representation and cortical folding for an evolutionarily old structure like V1 is species-specific and suggests potential differences in developmental and experiential constraints across primates.

**Supplementary Information:**

The online version contains supplementary material available at 10.1007/s00429-021-02427-0.

## Introduction

A major goal in systems and cognitive neuroscience is to understand the evolution of the human cerebral cortex (Van Essen 2007; Zilles et al. 2013). A central focus of this goal is to examine and quantify the correspondence between sulcal or gyral features relative to architectonically or functionally defined maps across different primate species (Van Essen 2007). Despite the widespread interest and a general convergence of conclusions across studies regarding comparisons of primary structures (e.g. the calcarine sulcus, CaS) and primary sensory areas (e.g. visual area V1; for reviews, see Rosa and Tweedale (2005), Van Essen (2007), Zilles et al. (2013), Arcaro and Kastner (2015), Van Essen et al. (2018), Van Essen and Glasser (2018), Wandell et al. (2007) and Wandell and Winawer (2011)), several historical observations have been commonly overlooked, which in turn, has generated modern discrepancies. Resolving these discrepancies through focused, cross-species comparative studies is critically necessary to produce accurate insights regarding the evolution of the cerebral cortex. As a majority (nearly 60–70%; Armstrong et al. 1995; Van Essen 2007; Van Essen et al. 2018) of the human cerebral cortex is buried in sulci, accurate insights regarding the coupling (or not) between sulci and functional maps are especially crucial.

**Fig. 1:**
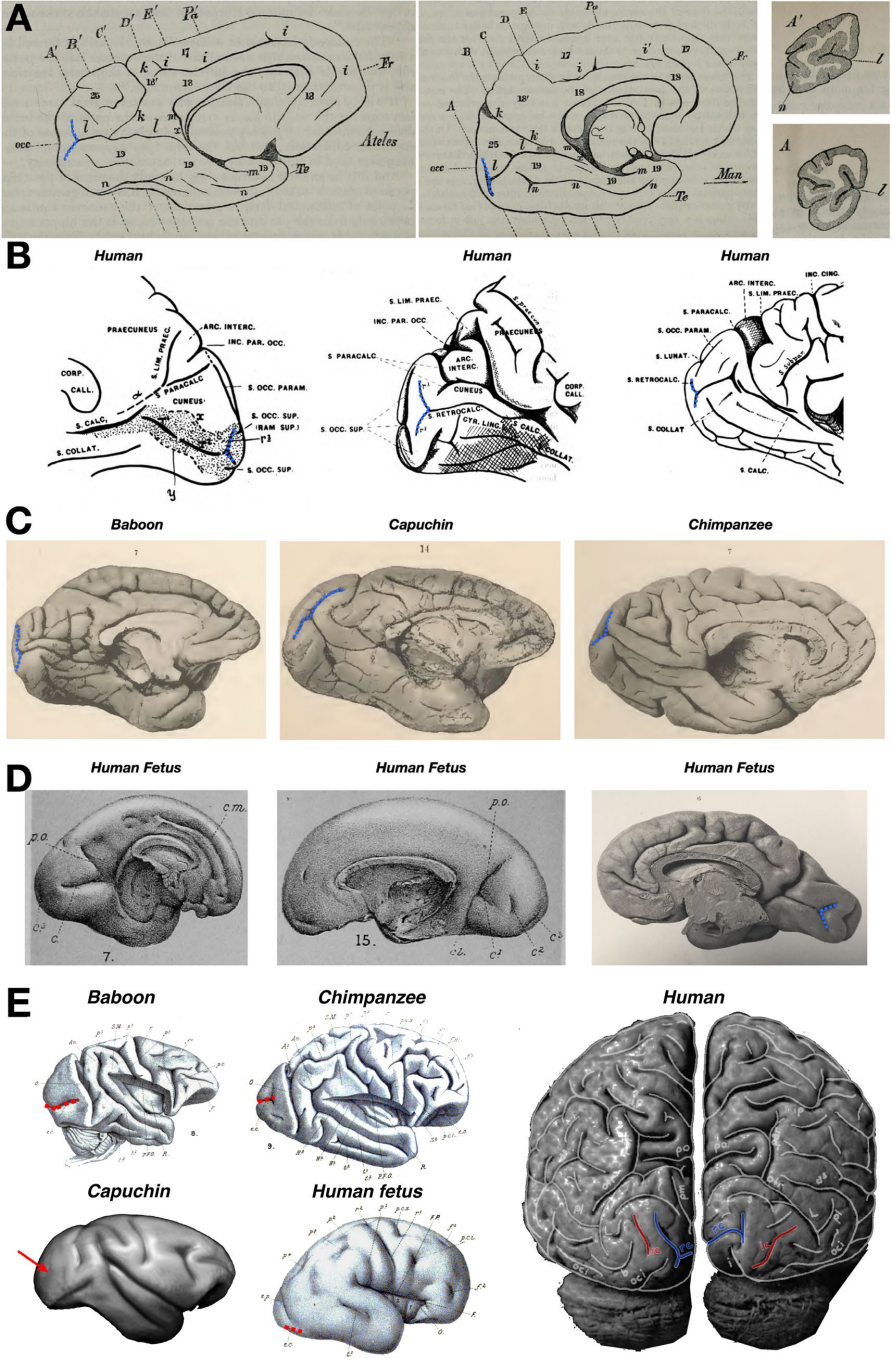
The retrocalcarine sulcus (rCaS) in the primate occipital cortex and in utero. **A** In the first labeling of the calcarine sulcus (CaS; *l* in each image) in 1861, Sir Thomas Huxley referred to the fact that there was a bifurcation (dotted blue line) in the posterior extent toward the occipital pole in both spider monkeys (*Ateles*; left) and humans (middle). Right: Coronal sections from *Ateles* (top; **A’**) and humans (bottom; **A**) in which the bifurcated portion of the posterior calcarine sulcus was described by Huxley. Images adapted from Huxley (1861). **B** Shortly after Huxley’s seminal observations, several labels were proposed for this posterior bifurcation of the CaS. For example, in a series of papers, Smith referred to this sulcus with several names such as sulcus retrocalcarinus verticalis (Smith 1902), the retrocalcarine sulcus (Smith 1904a), the sulcus occipitalis intrastriatus mesialis (retrocalcarinus) (Smith 1904b), or simply as r3 as depicted in the two leftmost images. Images adapted from Smith (1904a). **C** The rCaS (dotted blue line) is identifiable in several species included in the classic atlas by Retzius (1906). Left to right: baboon, capuchin, and chimpanzee. Images adapted from Retzius (1906). **D** Left and middle: drawings of two separate brains from early (left) or the middle (middle) of the 5th month of development. Cunningham referred to the rCaS as the posterior calcarine sulcus (c^3^ in the images). Images Adapted from Cunningham (1892). Right: A photograph of a human fetal brain from Retzius (1896). The rCaS (dotted blue) is easily identifiable, as is the external calcarine (eCaS; unlabeled), which is posterior to the rCaS. Images adapted from Retzius (1896). **E** The eCaS (dotted red line) is identifiable in several primate species in the atlas by Cunningham (1892) including baboons, chimpanzees, and humans. The external calcarine has not been identified in Capuchins, though a dimple is commonly found on the lateral surface in the approximate location where the external calcarine is found in Old World Monkeys (red arrow). The lack of a clear external calcarine, but presence of the retrocalcarine, in Capuchin monkeys indicates that these sulci emerged over different evolutionary timescales. Posterior view of adult human brain (Connolly 1950) shows the locations of both external and retrocalcarine sulci in red and blue lines, respectively

For example, in the very first labeling of the CaS in 1861, Huxley described a bifurcation in the posterior CaS in human and *Ateles* (spider monkey; Fig. 1A; Huxley 1861). Furthermore, in the late nineteenth and early twentieth centuries, this portion of the CaS was so frequently identified across species (Fig. 1) and was found to appear differentially in gestation compared to the CaS proper, that anatomists argued over distinct names for this vertical component of the CaS. For example, Cunningham (1892) referred to it eponymically as the vertical fissure of Sietz (1886) as well as the posterior calcarine sulcus (Cunningham 1892), while Smith suggested the term retrocalcarine (rCaS) sulcus (Smith 1903). Despite historical interest and contentions regarding this sulcus, there is still modern discrepancy regarding the existence of this bifurcation in humans. For example, while Iaria and colleagues (Iaria and Petrides 2007; Iaria et al. 2008) identified and quantified the morphological features of the rCaS in living and post-mortem human participants, Van Essen (2007), in the same year, stated that humans do not have this bifurcated sulcus:

“The calcarine sulcus also varies in shape, having a characteristic T-shaped posterior bifurcation in the macaque and chimpanzee that is lacking in humans.” (Van Essen 2007, pg. 271).

**Fig. 2:**
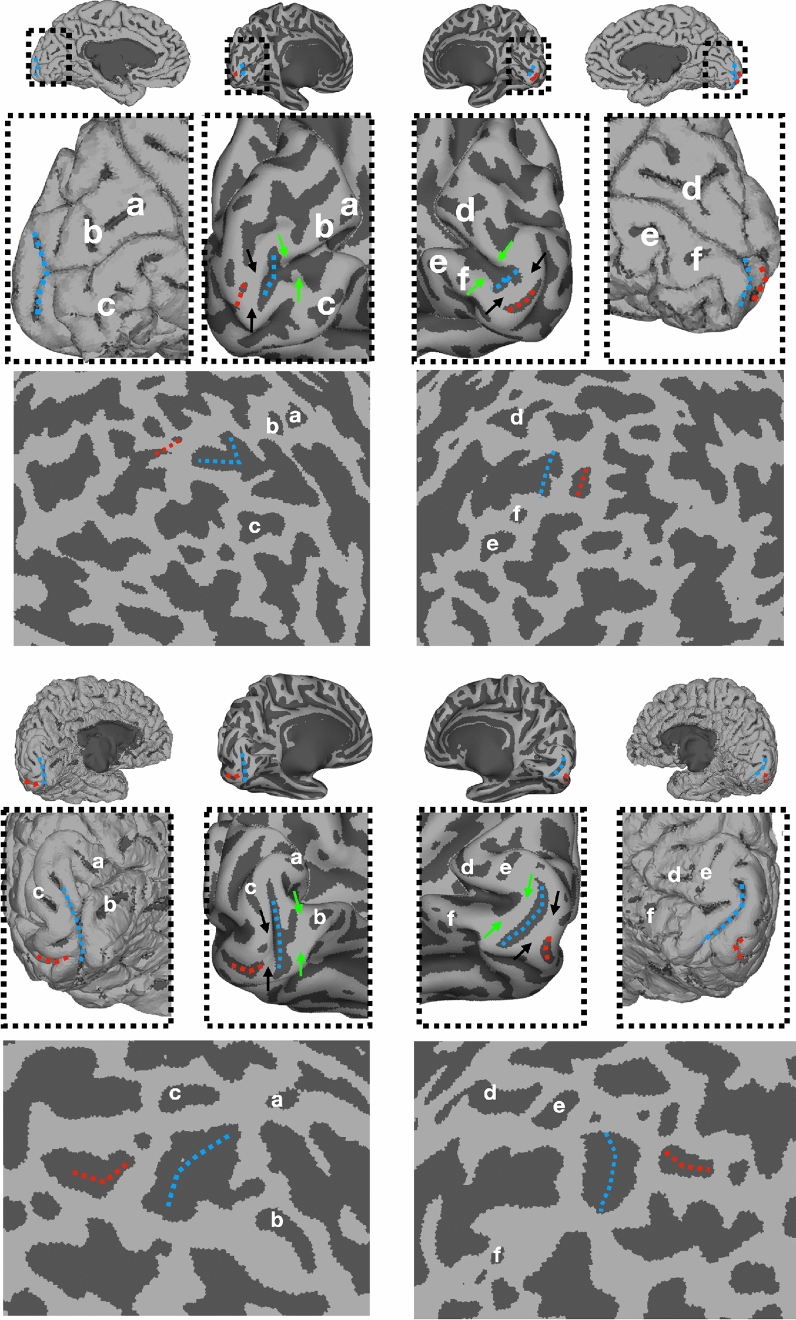
The rCaS is difficult to identify in flattened and inflated cortical surface visualizations. Medial views of the pial, partially inflated, and spherical surfaces from the left and right hemispheres of two example participants. HCP IDs: 690152 (top) and 145834 (bottom) randomly chosen from the 181 human participants included in the HCP 7 T Retinotopy Dataset (HCP7TRET; Benson et al. 2018). The labeling of sulci a-c and d-f in the left and right hemispheres, respectively, is aimed to guide the reader in identifying corresponding sulci across views. While the rCaS (dotted blue) is clearly visible on the pial surface, the flattening process often distorts the clear bifurcated morphology of the rCaS, which makes it hard to discriminate from the rest of the calcarine or the external calcarine sulci (red dotted lines). Arrows on partially inflated surfaces indicate previously identified “rungs,” or annectant gyri, across the calcarine by Schira and colleagues (2012) in which each rung has a predictable relationship with eccentricity. For example, the black arrows just anterior to the eCaS predict about 0.5° and the green arrows just anterior to the rCaS predict about 5° according to Schira and colleagues, which is consistent with our data (Figs. 5, 6 and 7)

We speculate that a main reason that cortical cartographers may not clearly identify the rCaS in humans is due to the way in which functional areas are typically defined in vivo using functional magnetic resonance imaging (fMRI). Common approaches for functional mapping of the human brain such as inflating or flattening a digitally reconstructed cortical surface provide utility by visualizing areas buried within sulci, which comprise the majority of cortex. However, the inflation and flattening process invariably distorts important features of the cortex. For example, while the rCaS is easily identifiable in post-mortem brains (Fig. 1) as well as the wrinkled (or pial) versions of cortical surface reconstructions, the inflation and flattening process can visually distort the rCaS to the point that it is not differentiable from the horizontal portion of the calcarine (Fig. 2).

**Fig. 3:**
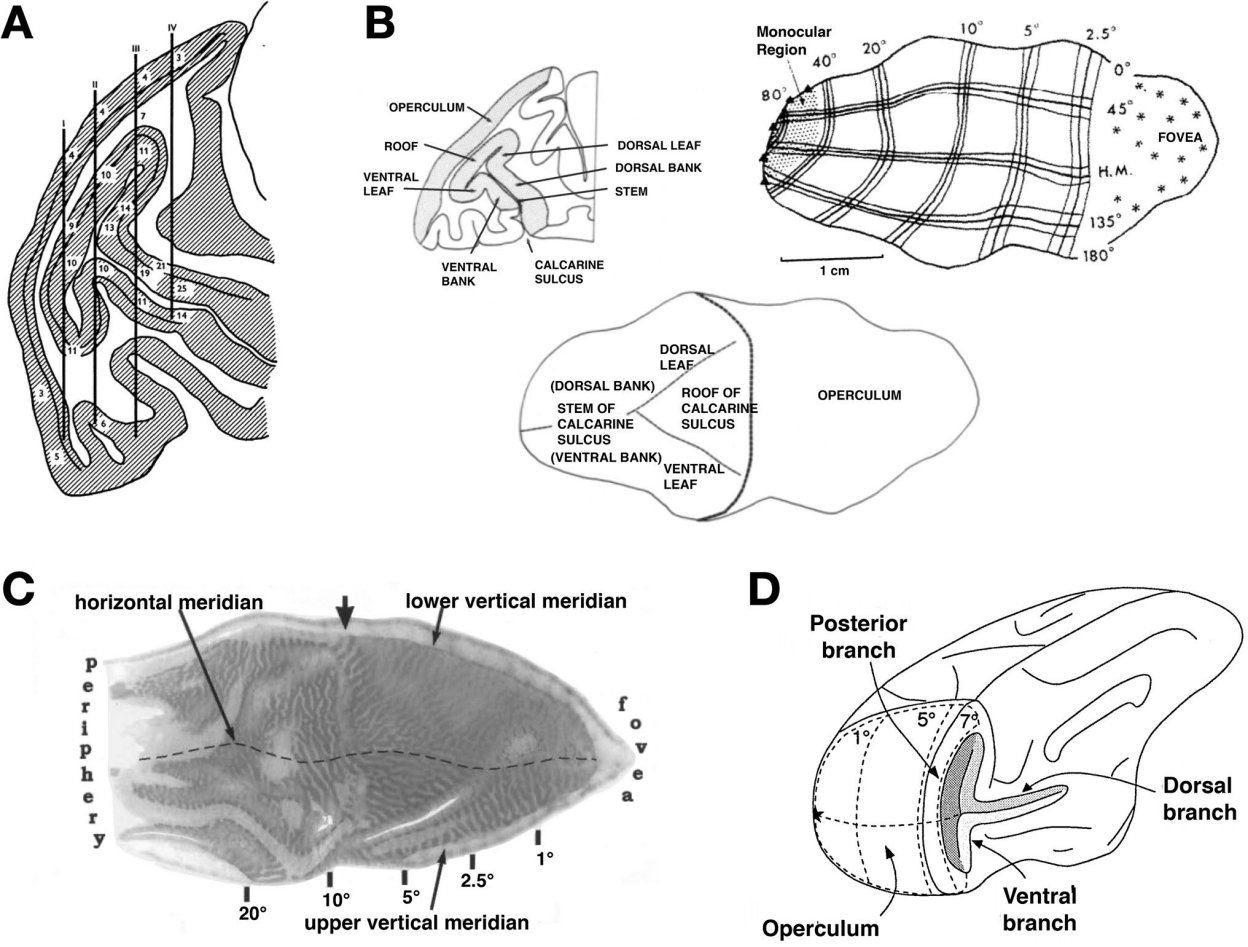
Mushrooms, roofs, leaves, hinges, and branches: eccentricity and the rCaS in non-human primates. **A** A drawing from Daniel and Whittredge (1961) of a baboon’s brain. Needle tracks (vertical black lines) are depicted relative to cortical locations that reflect the preferred neuronal firing to spots of light at a particular radial distance from the fixation point (numbers). The authors refer to the rCaS as a “mushroom” in which they write: “In sagittal section the calcarine cortex has the shape of a mushroom, with a ‘head’ and a ‘stem’. In sections further from the midline the 'head' gets smaller, and the most lateral sections show a ‘stem’ only, frequently cut obliquely (P1. 1; Text-Figs. 2, 3 And 7)” (pp. 207). **B** Left: outline of a parasagittal section from a macaque brain showing different parts of the rCaS (roof, ventral leaf, and dorsal leaf; Van Essen et al. 1984). The authors write, “Calcarine cortex has the configuration of a mushroom lying on its side, with a “stem” and a “head” each consisting of two sheets of cortex. The stem, to the left of the map, has dorsal and ventral banks joined along the fundus of the calcarine sulcus. The head of the mushroom has a “roof” and two “leaves” joined to the roof along separate branches of the Y-shaped fundus” (pp. 432). Right, bottom: drawings of flattened versions of V1 with labeled eccentricity values relative to the three pieces of the rCaS. Images adapted from Van Essen et al. (1984). **C** A flattened version of macaque V1 stained with cytochrome oxidase and labeled with eccentricity values, in which the arrow denotes the “hinge” of the operculum, which represents ~ 8°. The authors write: “The arrow denotes the “hinge,” where the operculum folds into the calcarine fissure at the midline” (p. 7230). Image adapted from Horton and Hocking (1996). **D** A drawing of a macaque brain, slightly rotated and labeled with eccentricity values in which 7° is just posterior to the rCaS, which consists of what is labeled as “posterior branch,” as well as the most posterior components of the “dorsal branch” and “ventral branch.” Image from Galletti et al. (2001)

Taking these methodological concerns into consideration, the present study compares eccentricity measurements in the rCaS and the nearby external calcarine sulci (eCaS) using fMRI in human participants (*n* = 24) and macaques (*n* = 6). We focused on eccentricity representations as classic electrophysiology studies have related eccentricity representations to the rCaS in macaques (Van Essen et al. 1984; Horton and Hocking 1996), reporting that the “hinge” of the operculum, which is just posterior to the rCaS, identifies ~ 7–8 degrees of eccentricity (Daniel and Whitteridge 1961; Van Essen et al. 1984; Horton and Hocking 1996; Galletti et al. 2001; Fig. 3). We also consider the eCaS because, like the rCaS, the eCaS is present in both species (“Materials and methods”). Our results show that despite similarities in the position of the eCaS on the lateral surface of the occipital lobe and the position of the rCaS on the medial surface of the occipital lobe in both species, the macaque rCaS comprises retinotopic representations 2–3 times further into the periphery than the human rCaS. In both species, the eCaS comprises retinotopic representations of central visual space with representations closer to the fovea in humans. Furthermore, average eccentricity representations are similar between the macaque eCaS and human rCaS. We discuss these findings in the context of understanding how the same macroanatomical structure across species could evolve different functional representations, as well as how functionally homologous areas across species can potentially differ in their underlying anatomical substrates.

## Materials and methods

### Participants

#### Humans

Twenty-four adults (ages 22–35, 14 female) were randomly selected from the 181 participants comprising the HCP 7 T Retinotopy Dataset (HCP7TRET; Benson et al. 2018). All participants had normal or corrected-to-normal visual acuity. Each participant was assigned a six-digit HCP ID.

#### Macaques

Six juvenile Macaca mulattas (three female) participated in this study. Four monkeys were selected from (Arcaro and Livingstone 2017a). All procedures were approved by the Harvard Medical School Animal Care and Use Committee and conformed with National Institutes of Health guidelines for the humane care and use of laboratory animals. For scanning, monkeys were alert and their heads were immobilized using a foam-padded helmet with a chinstrap that delivered juice. The monkeys were scanned in a primate chair that allowed them to move their bodies and limbs freely, but their heads were restrained in a forward-looking position by the padded helmet.

### Stimuli

For both human and macaque experiments, visual stimuli were projected onto a screen at the end of the scanner bore.

#### Human retinotopic mapping

In the HCP7TRET experiment, retinotopic mapping stimuli were constructed by creating slowly moving apertures consisting of rotating wedges, expanding/contracting annuli, and oriented bars constrained to a circular region with a diameter of 16**°** centrally presented on the screen. Dynamic colorful textures composed of objects on an achromatic pink-noise background were placed within the apertures. Apertures were animated at 15 Hz. Each run lasted 300 s with a total of 6 runs per participant. Participants were instructed to maintain fixation on a centrally presented dot and to perform a color change detection task. See Benson et al. (2018) for more details.

#### Macaque retinotopic mapping

Retinotopic mapping stimuli were constructed by creating slowly moving apertures consisting of rotating wedges and expanding/contracting annuli constrained to a circular region with a diameter of 20**°** centrally presented on the screen. Dynamic colorful checkerboards filled the apertures in which each check’s chromaticity and luminance alternated at the flicker frequency of 4 Hz. Polar angle (wedge) and eccentricity (annulus) mapping were conducted in separate experiments each consisting of 8–12 runs with an equal split in the direction of rotation. Each polar angle run consisted of eight cycles lasting 40 s each. Each eccentricity run consisted of seven cycles lasting 40 s each with 10 s of blank, as well as black backgrounds in between. These additional blank periods were inserted to temporally separate responses to the foveal and most peripheral positions. The monkeys were rewarded with juice for maintaining a central fixation within a 2**°** window. Gaze direction was monitored using an infrared eye tracker (ISCAN, Burlington, MA). See Arcaro and Livingstone (2017a) for more details.

### Data acquisition

Human data were collected at the Center for Magnetic Resonance Research at the University of Minnesota using a Siemens 7T Magnetom actively shielded scanner and a 32-channel received coil array with a single channel transmit coil (Nova Medical, Wilmington, MA). Macaque functional data were collected in a 3T Siemens TimTrio scanner with an AC88 gradient insert using 4-channel surface coils (custom made by Azma Mareyam at the Martinos Imaging Center). Macaque anatomical data were collected in a 3T Siemens Skyra scanner using a 15-channel transmit/receive knee coil.

#### Human anatomical scans

T1-weighted (T1w) and T2-weighted (T2w) structural scans at 0.7-mm isotropic resolution were acquired at 3T and used as the anatomical substrate for the retinotopy data. See Benson et al. (2018) for full details.

#### Human functional scans

Whole-brain scans were collected using a T2*-sensitive gradient echo planar pulse sequence at a resolution of 1.6 mm isotropic and 1 s TR (multiband acceleration 5, in-plane acceleration 2, 85 slices). See Benson et al. (2018) for full details.

#### Macaque anatomical scans

A whole-brain structural volume was acquired, while the animals were anesthetized with a combination of Ketamine (4 mg/kg) and Dexdomitor (0.02 mg/kg). Monkeys were scanned using a T1w magnetization-prepared rapid gradient echo (MPRAGE) sequence; 0.5 × 0.5 × 0.5 resolution; FOV = 128 mm; 256 × 256 matrix; TR = 2700 ms; TE = 3.35 ms; TI = 859 ms; flip angle = 9°). 3 whole-brain anatomical images were collected in each animal.

#### Macaque functional scans

Whole-brain scans were collected using a T2*-sensitive gradient echo planar pulse sequence at a resolution of 1 mm isotropic and 2 s TR (in-plane acceleration 2, 67 slices). To enhance contrast (Vanduffel et al. 2001), we injected 12 mg/kg mono-crystalline iron oxide nanoparticles (Feraheme, AMAG Pharmaceuticals, Cambridge, MA) in the saphenous vein just before scanning. See Arcaro and Livingstone (2017a) for full details.

### Data analyses

Data were analyzed using Human Connectome Workbench, Analysis of Functional NeuroImages (AFNI), SUMA, FreeSurfer (FreeSurfer; Fischl et al. 1999a, b), JIP Analysis Toolkit (written by Joseph Mandeville), and MATLAB (Mathworks).

#### Reconstruction of human cortical surfaces

White and pial cortical surfaces were reconstructed from the structural scans using the HCP Pipelines (Glasser et al. 2013). Surfaces were aligned across participants to the HCP 32 k fs_LR standard surface space using a twofold approach. First, a gentle folding-based registration was used (referred to as ‘‘MSMSulc’’). Second, a more aggressive areal feature-based registration was used (referred to as ‘‘MSMAll’’). The latter approach is multi-modal in nature and uses myelin maps, resting-state network maps, and 3T resting-state visuotopic maps (Robinson et al. 2014, 2018; Glasser et al. 2016) to reconstruct cortical surfaces and align one to another.

#### Reconstruction of macaque cortical surfaces

Each animal’s three anatomical scans were co-registered and averaged. Each average structural volume underwent semi-automated cortical surface reconstruction using FreeSurfer. To ensure high accuracy, skull stripping and white matter masks were first manually segmented by an expert (MJA) then passed into FreeSurfer’s autosegmentation pipeline. If poor segmentations were detected, the white matter mask and control points were edited, and the surface reconstruction was rerun until corrected. To fix segmentation errors, average anatomical volumes were manually edited to improve the grey/white matter contrasts and to remove surrounding non-brain structures (e.g., sinuses, arachnoid, and dura matter). See Arcaro and Livingstone (2017a) for more details.

#### Human functional data analyses

The data were processed using the HCP pipelines as previously published (Glasser et al. 2013), which correct for head motion and EPI distortion and register the functional data to individual participant surfaces. The time series data were analyzed using a population receptive field (pRF) model referenced as the Compressive Spatial Summation model (Kay et al. 2013; http://cvnlab.net/analyzePRF).

#### Macaque functional data analysis

The data were processed using AFNI. All images from each scan session were motion corrected and aligned to a single timepoint for that session. Data were detrended and spatially filtered using a Gaussian filter of 2 mm full-width at half-maximum (FWHM) to increase the signal-to-noise ratio (SNR), while preserving spatial specificity. Data were registered using a two-step linear then non-linear alignment approach (JIP analysis toolkit) to a standard anatomical template for all monkeys. Fourier analysis was used to identify spatially selective voxels from polar angle and eccentricity experiments. Results from this analysis approach were originally reported in Arcaro and Livingstone (2017a) and are further quantified in this paper.

### Sulcal definitions

#### Classic and modern definitions of the rCaS

As described in the Introduction, while there is modern discrepancy regarding the identification of the rCaS in humans, the posterior bifurcation of the CaS was first identified across species in 1861 by Huxley and has continued to be identified by both classic and modern neuroanatomists (Fig. 1). Here, we include some classic and modern quotes describing this posterior bifurcation and the rCaS. For example, in describing the posterior extent of the CaS in humans, Huxley (1861) wrote:

“Traced from before backwards, or from within outwards, the line of this sulcus presents a strongly marked, but irregular, upward convexity.

On making successive transverse sections of this cerebrum from before backwards (woodcut, Fig. 1A–D), the fissure was seen, in its most posterior part (A), to pass almost horizontally outwards for a short distance, and then to divide into an upward and a downward branch.” (pgs. 254–255).

When describing the posterior bifurcation of the CaS in *Ateles*, Huxley (1861) wrote: “The calcarine sulcus, l, l, has the same general direction and the same bifurcated termination, as in Man.” (pg. 256).

Just over three decades later, Cunningham (1892) referred to this bifurcation of the CaS as the posterior calcarine sulcus, as well as eponymically as the vertical fissure of Sietz (1886). Even though Cunningham references Sietz’ work, we highlight that Sietz did not label this sulcus as the retrocalcarine or the posterior calcarine, and instead, referred to this sulcus as “Endfurche, F. extrema” ((Sietz 1886), pg. 275; see Supplementary Fig. 1 for images from Sietz (1886)).

Furthermore, Smith (1904a) credited Cunningham as the “first writer to draw any distinction between the calcarine and the retrocalcarine sulci. His reason for doing so was, briefly, the fact that the latter sulcus developed later and independently of the calcarine” (Smith 1904a, pg.128). Cunningham, however, did not use the retrocalcarine nomenclature. He referred to this sulcus as fissure calcarina posterior. Smith (1903) proposed the retrocalcarine name:

“The sulcus which Cunningham calls “posterior calcarine” develops later and quite independently of the anterior sulcus; it never becomes as deep as the former; as a rule it does not share in the formation of the calcar, and in many cases, it is separated from the anterior or calcarine sulcus by a submerged fold of cortex. It is, to use Cunningham’s own words, “a secondary sulcus in every sense of the term.” It is, therefore, of a very different nature to the true calcarine sulcus, and, as it is convenient to have a distinctive name, I shall call it “retrocalcarine,” because it is placed on the caudal side of the calcar…This has been done, not for pedantic reasons, but because a separate name becomes absolutely necessary in Comparative Anatomy, where the fundamental distinction between the two elements becomes more pronounced” (Smith 1903, pg. 386).

In more modern work, Duvernoy (1999) defines the retrocalcarine in a similar fashion to these historical definitions:

“The calcarine sulcus is often terminated by the retrocalcarine sulcus. A small gyrus—the gyrus descendens of Ecker (Fig. 8)—is posteriorly bounded by the retrocalcarine sulcus and anteriorly by the variable occipitopolar sulcus. On the lateral surface, the boundary of the striate area is delineated by the gyrus descendens.” (pg. 16).

It should also be noted that Duvernoy denotes different labels for the bifurcated branches, or rami, of the retrocalcarine sulcus as the inferior (pgs. 214, 216) and superior (pg. 214) retrocalcarine sulci.

Likewise, in the most recent atlas to define the rCaS in the human brain, Petrides (2019) also defines the rCaS in a consistent manner relative to these previous definitions. Petrides (2019) writes: “The retrocalcarine sulcus (i.e. the tail of the calcarine sulcus) can be observed at the occipital pole and the cortex that spreads around it” (pg. 84).

Guided by these definitions, we defined the rCaS in humans and macaques as the two bifurcated components of the posterior CaS positioned on the medial side of the occipital pole.

#### Classic and modern definitions of the eCaS

The external calcarine sulcus (eCaS) is very prominent on the lateral surface of the occipital lobe in macaque (and it is sometimes referred to as the ectocalcarine sulcus; Sinich et al. 2003; Yeterian and Pandya 2010; Distler et al. 1993), but is much smaller in human. Specifically, the human eCaS can be one or a series of smaller sulci located toward the occipital pole positioned dorsally to the occipito-polar sulci (Petrides 2019). Furthermore, classic and modern anatomists (Smith 1904a, b; Connolly 1950; Duvernoy et al. 1992; Petrides 2019) credit Cunningham (1892) with the labeling of the external calcarine. For example, Smith (1904a) wrote:

“When it is recalled that the sulcus occipitalis superior occupies a position (within the area striata) on the lateral surface analogous to that of the retrocalcarine (Cunningham’s “posterior calcarine”) on the mesial surface it will be apparent that Cunningham’s term “external calcarine” is not inappropriate as a designation for the former furrow. Moreover, the gradual slipping over of the area striata lateralis on to the mesial surface in the human brain must imply that the homologue of that caudal part of the lateral stria-bearing cortex, which is folded to form the posterior part of the superior occipital (C’s external calcarine) sulcus in the Apes, will in the human brain form the walls of the caudal part of the sulcus retrocalcarinus (C’s posterior calcarine).” (pgs. 134–135).

Connolly (1950) also wrote:

“The term external calcarine given by Cunningham or its equivalent the lateral calcarine used by Ingalls (1914) appears to be the most suitable as it is a part of the calcarine complex, and like the posterior or retrocalcarine, is axial to the striate area.” (pg. 8).

As reflected in the above quotation, Connolly referred to the eCaS as the lateral calcarine in his atlas across species (Fig. 1E). He also referenced a dorsal ramus of this sulcus, which is sometimes evident in our data (Fig. 5, dark grey sulcal fold on lateral views of macaque surfaces extending from the foveal confluence to the posterior opercular tip near yellow and green asterisks). Connolly (1950) wrote:

“Beginning just above the lower end of the lunate sulcus, the main constituent of the lateral calcarine (lc) extends obliquely backward to the occipital pole. Just above it is its detached dorsal ramus.” (pg. 27).

Finally, in the most recent atlas to define the eCaS, Petrides (2019) refers to the “external calcarine sulcus (sulcus calcarinus externus of Cunningham)” (pg. 29).

Following these classic and modern definitions of the eCaS, we defined the eCaS in all 48 hemispheres in humans (Supplementary Fig. 2) and 12 hemispheres in macaques relative to the rCaS.

#### Manual identification of rCaS and eCaS

In humans, rCaS and eCaS were manually defined by a neuroanatomist (KSW) in each individual based on the classic and modern definitions described above. In macaques, rCaS and eCaS were also identified by a neuroanatomist (KSW) guided by the work described in the previous section, as well as additional modern studies (Van Essen et al. 1984; Horton and Hocking 1996; Galletti et al. 2001; Fig. 3) and then manually defined by MJA.

### Functionally defined V1

Smooth, continuous representations of visual space were identified along the cortical surface for both polar angle and eccentricity mapping experiments in each individual human and macaque (Fig. 5; Supplementary Fig. 3). The border between V1 and V2 was identified by reversals in polar angle phase progression at the lower (Fig. 5; blue colors in angle maps) and upper (red colors) visual fields in dorsal and ventral portions of the CaS, respectively, and extending onto the lateral surface of the occipital lobe to a variable extent between species (much more so in macaques than humans). Specifically, the lateral-medial extent of V1 was identified by a progression from the most foveal (Fig. 5; red colors in eccentricity maps) to the most peripheral (blue colors) representations.

### Cortical surface measurements

#### Cortical surface area

For rCaS and eCaS, cortical surface area was measured along the pial and (smoothed) white matter surface segmentations using AFNI’s SurfMeasures. Surface area was measured both in raw units (mm^2^) and normalized to the total surface area of V1 in each participant (Supplementary Fig. 4).

#### Distance from V1 foveal confluence

Cortical distance between each point (surface node) within V1 and the foveal confluence was estimated along pial surface segmentations using AFNI’s SurfDist. Because the vertical meridian boundary between V1 and V2 is difficult to measure within the fovea using fMRI, the foveal confluence was defined as a curved line through the most foveal measurements linking the upper and lower vertical meridian boundaries identified within the surrounding parafoveal regions. Cortical distance from the fovea was defined as the minimum Euclidean distance along the cortical surface between each V1 surface node and the foveal confluence line. Treating the foveal confluence as a single point defined at the midpoint along this line yielded qualitatively similar results for subsequent analyses and did not change interpretation of the data. We focused on eccentricity representations as classic electrophysiology studies have related eccentricity representations to the rCaS in macaques (Van Essen et al. 1984; Horton and Hocking 1996), reporting that the “hinge” of the operculum, which is just posterior to the rCaS, represents ~ 7°–8° (Daniel and Whitteridge 1961; Van Essen et al. 1984; Horton and Hocking 1996; Galletti et al. 2001; Fig. 3).

#### Cortical magnification

For each participant, eccentricity measurements within V1 were plotted as a function of cortical distance from the foveal confluence. This produced a scatter plot in which each data point represents a single surface node (Fig. 6). An exponential curve as proposed in Strasburger (2019; Eq. 16) was then fit to the data points:

$$E = a\left( {e^{{b\hat{d}}} - 1} \right),$$

where *E* is the predicted eccentricity (in °), *d̂* = cortical distance, and *a* and *b* are free parameters. The constant term (− 1) allows for fitting foveal (< 1°) measurements. The curve was fit using MATLAB’s Curve Fit Tool, minimizing the sum of the squared errors between the actual eccentricity values and the eccentricity values predicted by the curve.

To further illustrate the relationship of the rCaS and eCaS to the cortical magnification of V1 in humans and macaques, we computed a 2D histogram using bins of 0.5° eccentricity and 1 mm cortical distances for all datapoints in V1. We then used MATLAB’s contour function to identify isolines positioned at 99, 75, 50, and 25% of the maximum bin for data within the rCaS and eCaS separately.

### Visual field coverage

The average eccentricity representation within both rCaS and eCaS was calculated for each participant. The group mean and standard error across participants were calculated. Polar angle and eccentricity measurements for each surface node were converted to Cartesian space (Matlab’s pol2cart) and visualized in scatter plots for humans and macaques separately.

### Statistical analyses

We evaluated visual field coverage and areal size differences across species using two-way ANOVAs with species (humans/macaques) and hemisphere (right/left) as factors.

## Results

**Fig. 4:**
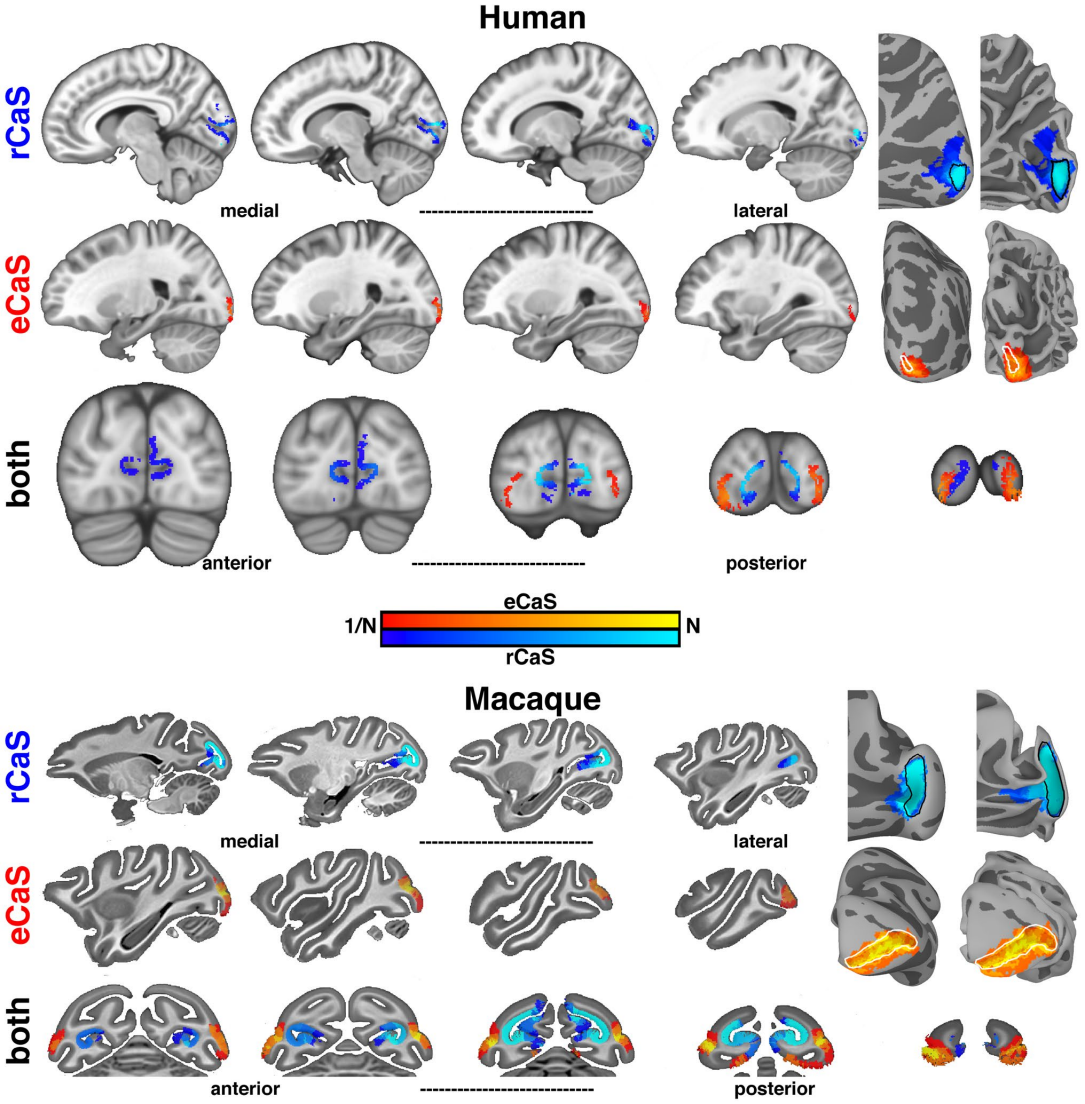
Anatomical localization of the rCaS and eCaS in human and macaque. (Left) Sagittal and coronal slices of group overlap maps for rCaS (blue) and eCaS (red) for (top) humans and (bottom) macaques. Group overlap maps range from most (bright colors) to least (dark colors) overlap. Sagittal and coronal human slices are spaced every 3 mm and 4 mm, respectively. Sagittal and coronal macaque slices are spaced every 2.5 mm. (Right) Group overlap maps for rCaS and eCaS shown on inflated and pial cortical surface views of human (fsaverage; top; Fischl et al. 1999a, b) and macaque (NMT; bottom; Seidlitz et al. 2018) template surfaces. Outlines of the rCaS (black solid line) and eCaS (white solid line) defined from the folding patterns of the template surfaces for both species are shown for inflated and folded views. Colormap ranges from 1/*n* to *n* individuals

The retrocalcarine (rCaS) and external calcarine (eCaS) sulci were identified bilaterally in 24 humans and 6 macaques. In both species, the rCaS and eCaS were localized to the medial and lateral surfaces of occipital cortex, respectively. In both macaques and humans, the rCaS was located on the most posterior portion of the medial surface with the long axis oriented along the inferior-superior dimension. The macaque eCaS was located in the ventral half of the operculum of the occipital lobe with the long axis oriented along the posterior–anterior axis. The human eCaS was identified as one or a series of smaller sulci located toward the occipital pole positioned dorsally to the occipito-polar sulci (Petrides 2019). To examine the consistency and variability in the cortical position of these sulci across individuals, each individual’s native surfaces were aligned to species-specific (human: FreeSurfer (Fischl et al. 1999a, b); macaque: NMT (Seidlitz et al. 2018)) template surfaces (Fig. 4). Group overlap maps were calculated across individuals and compared to the rCaS (black lines) and eCaS (white lines) identified on each species-specific group template. There was considerable overlap across individuals for both sulci with no confusability between sulci. For example, variability in the rCaS most often occurred anteriorly and superiorly, but remained on the medial surface, while variability in the eCaS most often occurred ventrally and anteriorly, but did not extend to the medial occipital surface. The group overlap maps were also projected into volumetric space for visual reference (Fig. 4). The presence of the rCaS and eCaS in each individual confirms that these sulci are prominent macroanatomical structures in both humans and macaques.

In macaques, both the rCaS and eCaS fell within V1 in each individual. In humans, the rCaS fell within V1 in each individual, but the eCaS fell outside of V1 for several participants (9 of 48 hemispheres). In both species, the surface area of the rCaS (average human: 279.68 mm^2^ ± 39.01; average macaque: 187.91 mm^2^ ± 12.07) was larger than the eCaS (average human: 72.60 mm^2^ ± 9.36; average macaque: 82.45 mm^2^ ± 5.67) and comprised a larger portion of V1’s total surface area (Supplementary Fig. 4; average human: 14.35% ± 1.58 vs. 1.77% ± 0.21; average macaque: 19.09% ± 0.56 vs. 8.18% ± 0.58). Notably, the size of the eCaS relative to V1 was substantially larger in macaques than humans (significant main effect of species: *F*(1,56) = 158.36, *p* < 0.001; no significant effect of hemisphere and no interaction, *p* > 0.05). The size of the rCaS relative to V1 was also larger in macaques than humans (significant main effect of species: *F*(1,56) = 8.38, *p* < 0.006; no significant effect of hemisphere and no interaction, *p* > 0.05).

**Fig. 5:**
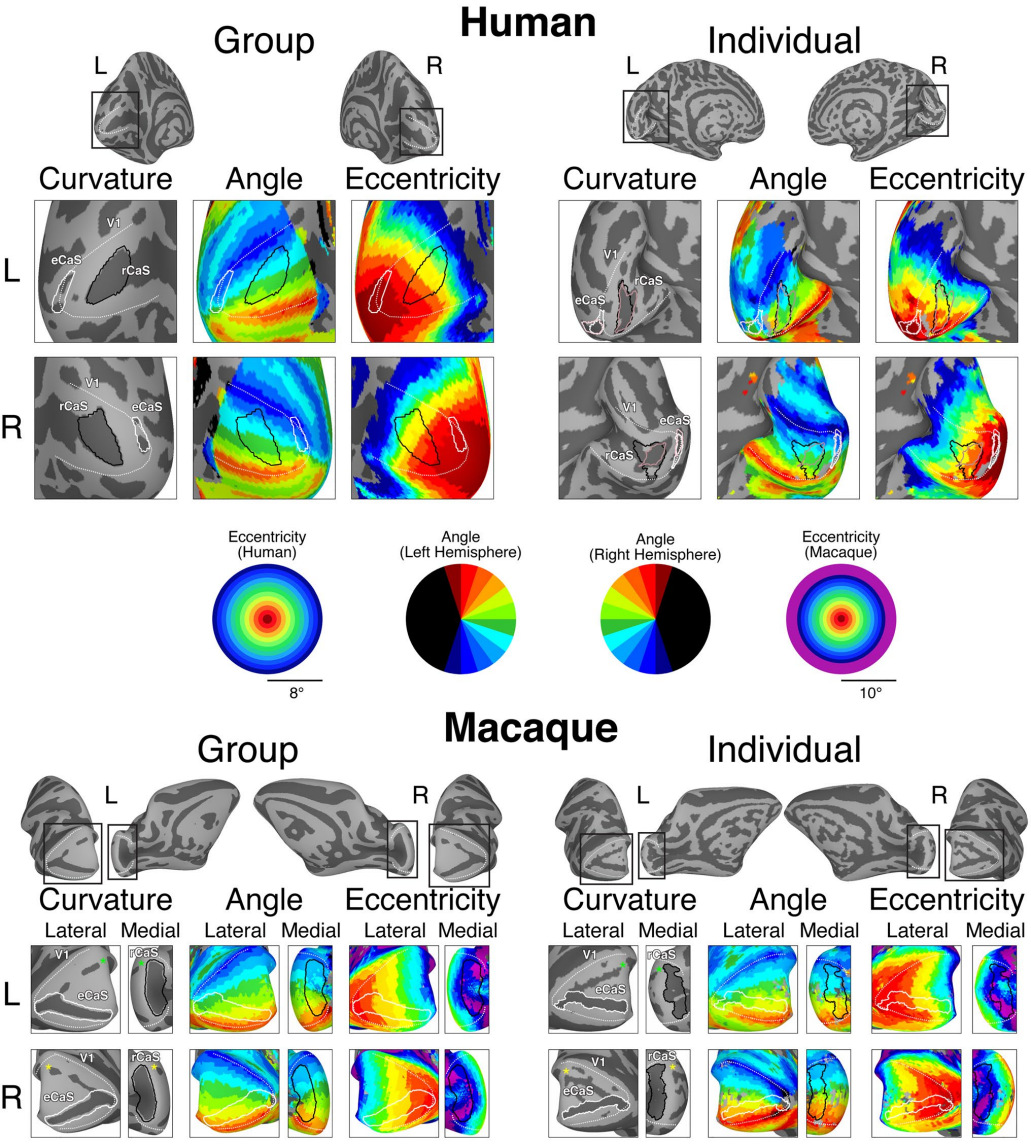
The rCaS and eCaS relative to V1 in humans and macaques. Outlines of template-defined rCaS (black solid line) and eCaS (white solid line) and individual-defined rCaS (dark pink solid line) and eCaS (light pink solid line) on cortical surface curvature, polar angle, and eccentricity maps in humans (top) and macaques (bottom). Group averaged (left) and individual participant (right: 144226 and M1 for human and macaque, respectively) data are shown. White dotted lines illustrate the borders between visual areas V1 and V2. To help relate the lateral and medial viewpoints of the macaque surfaces, green and yellow asterisks mark corresponding locations in V1. Zoomed out views are shown for each hemisphere with black boxes corresponding to the region shown in the cropped images. See Supplementary Fig. 3 for more example participants

**Fig. 6:**
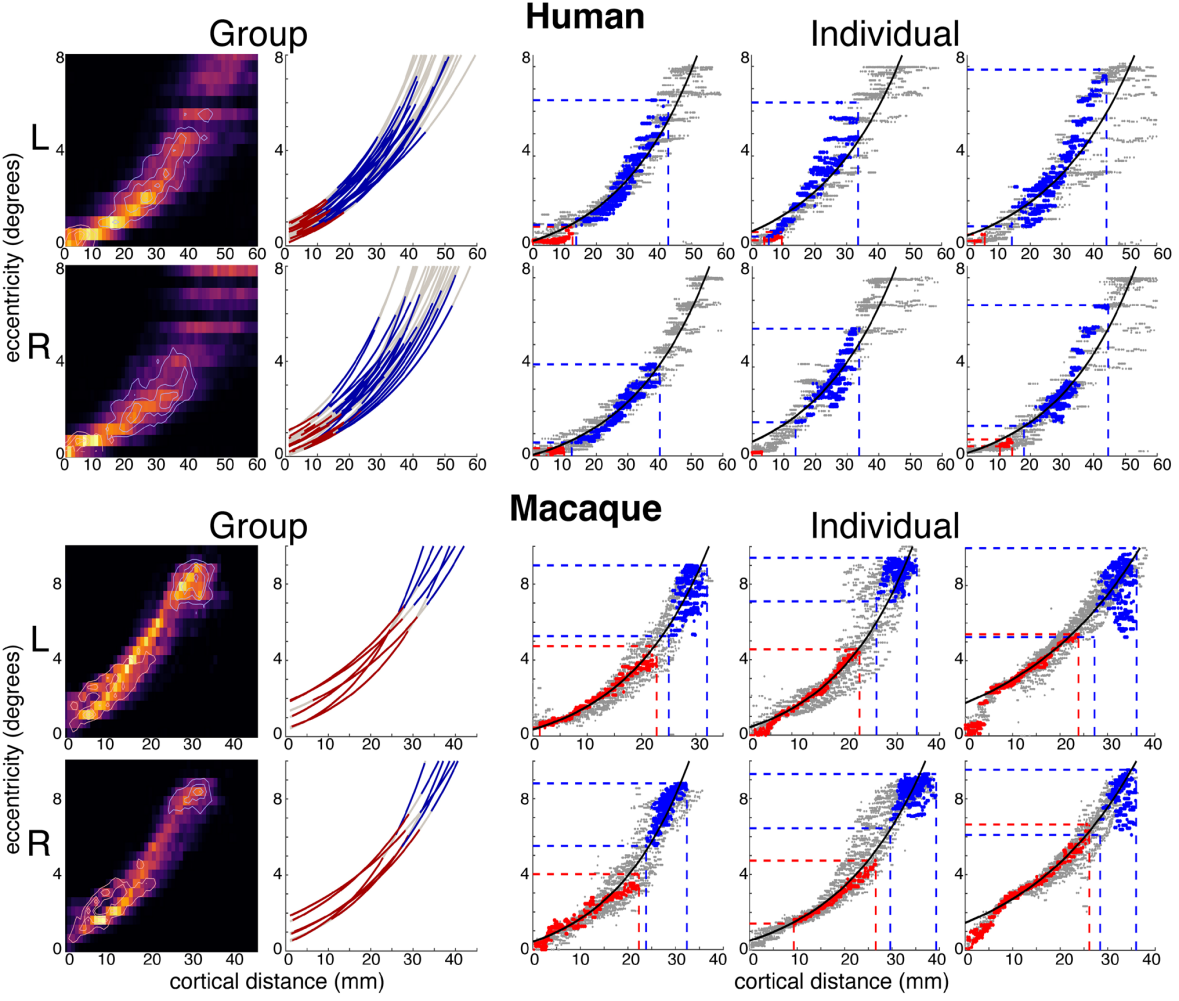
Eccentricity representations of rCaS and eCaS as a function of cortical distance in humans and macaques. The location of eCaS and rCaS in relation to the cortical magnification of V1 are shown for (left) group and (right) individual participants in (top) humans and (bottom) macaques. (Left) Group aggregated 2D histograms and exponential curve fits to eccentricity as a function of cortical distance from the foveal confluence of V1. For histograms, isocontour lines are shown for both eCaS and rCaS at 99, 75, 50, and 25% levels of their respective maximums. For exponential curve fits, the average *r*-squared for individual fits was 0.89 (± 0.02) and 0.95 (± 0.02) for humans and macaques, respectively. While the curve fits do not capture all aspects of the data, they provide an accurate illustration of the relationship between eccentricity and cortical distance for both eCaS and rCaS. The range of eccentricity and cortical distances covered by the rCaS (blue line) and eCaS (red line) are shown relative to the rest of V1 (grey line). (Right) Scatterplots of eccentricity representations in relation to cortical distance from the fovea of V1 within the rCaS (blue), the eCaS (red), and the rest of V1 (grey) for three individuals (left to right: 100610, 102816, 114823 and M1, M2, M3 for humans and monkeys, respectively). Black lines illustrate the curve fits across the entire V1. The range of eccentricity and cortical distances covered by the rCaS and eCaS are illustrated by red and blue dashed lines, respectively. See Supplementary Fig. 5 for additional example individuals

**Fig. 7:**
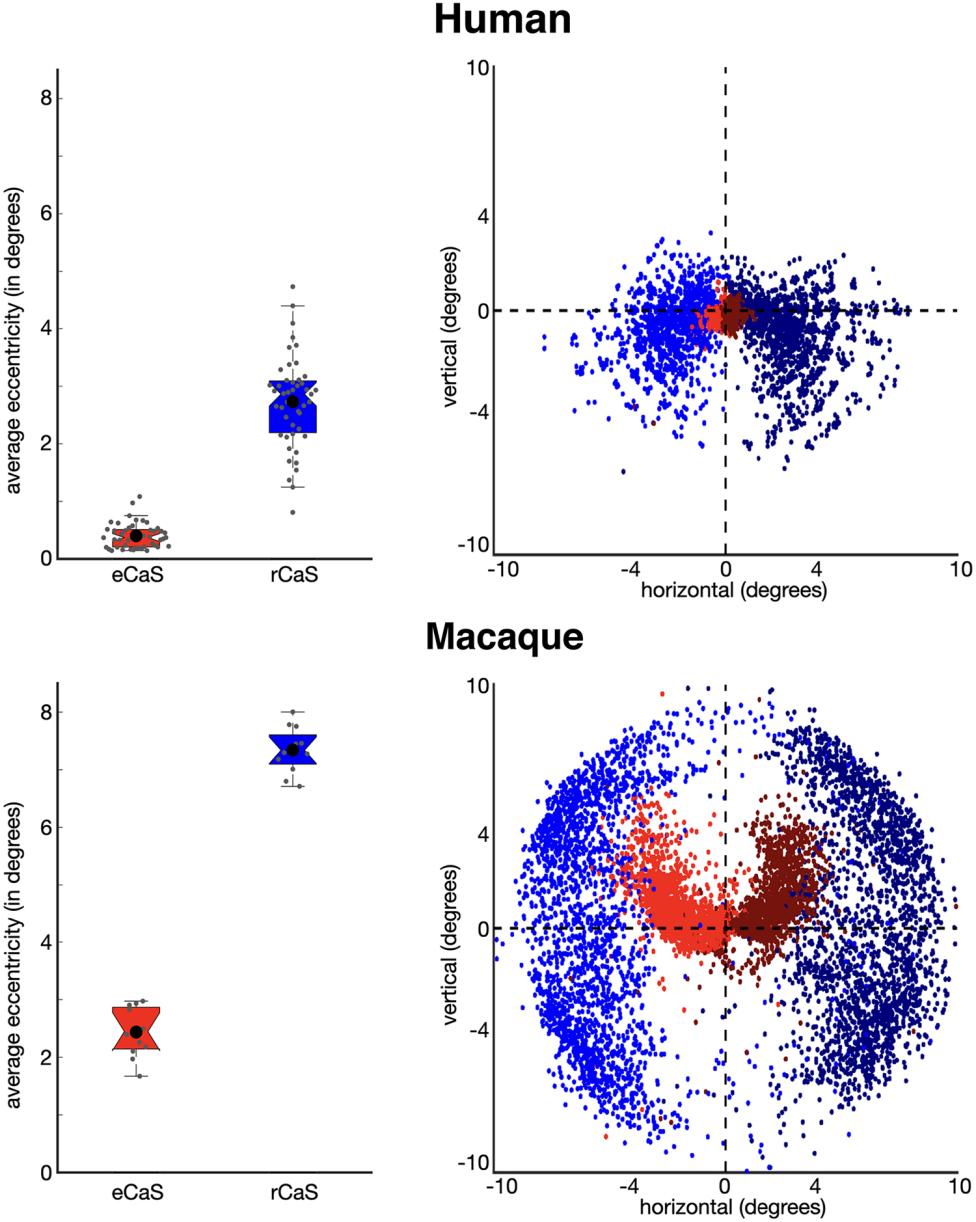
Visual field coverage of rCaS and eCaS in humans and macaques. (Left) The mean eccentricity representation within the rCaS (blue) and eCaS (red) for individuals (grey circles) and group averages (black circles) in humans (top) and macaques (bottom). (Right) Visual field coverage in Cartesian space of each surface node within the rCaS and eCaS across all participants

In both species, the rCaS and eCaS were localized to specific parts of the eccentricity map in V1 (Figs. 5, 6 and 7). Specifically, the eCaS preferred representations occupied by central visual space and the rCaS preferred representations occupied by more eccentric locations of visual space. For both the rCaS and eCaS, the average visual field representations differed between species. Across participants, the mean eccentricity representation of the eCaS was 0.40**°** (± 0.03) in humans and 2.45° (± 0.12) in macaques (significant main effect of species: *F*(1,56) = 551.34, *p* < 0.0001; no significant effect of hemisphere and no interaction, *p* > 0.05). Across participants, the mean eccentricity representation of the rCaS was 2.73° (± 0.11) in humans and 7.34° (± 0.06) in macaques (significant main effect of species: *F*(1,56) = 403.5, *p* < 0.0001; no significant effect of hemisphere and no interaction, *p* > 0.05). Differences in visual field coverage between humans and macaques were apparent by visualizing the range of eccentricity representations within the eCaS, within the rCaS, and across the rest of V1 as a function of cortical distance from the foveal confluence (Fig. 6). The eCaS covered a wider range of eccentricities in macaques than humans (significant main effect of species: *F*(1,47) = 725.37, *p* < 0.0001; no effect of hemisphere or interaction, *p* > 0.05; Fig. 6, Supplementary Fig. 5; red dots and lines). The rCaS, on the other hand, covered a wider range of eccentricity representations in humans compared to macaques (significant main effect of species: *F*(1,56) = 10.82, *p* < 0.002; no significant effect of hemisphere or interaction, *p* > 0.05; Fig. 6, Supplementary Fig. 5; blue dots and lines). These species differences in visual field coverage for eCaS and rCaS reflect a general shift of V1’s map to the medial surface in humans as well as relative size differences of eCaS between species. In macaques, the central 7°–8° (Fig. 5, red to cyan colors in eccentricity maps) are located on the lateral (opercular) surface. In humans, almost the entire V1 map is located on the medial surface with only the fovea (Fig. 5, red in eccentricity maps) extending onto the lateral surface. This medial shift is also reflected by similar average eccentricity values within different sulci between species. That is, the eCaS on the lateral surface in macaques has a similar average eccentricity value (mean = 2.45 ± 0.12) as the rCaS in humans (mean = 2.73 ± 0.11; no main effect of species, hemisphere, or interaction, *Fs*(1,55) < 1.67, *ps* > 0.201).

Finally, visual field coverage with respect to polar angle also differed between species for both sulci (Fig. 7, right). In humans, the eCaS spanned upper and lower visual field representations with, if anything, a slight bias for lower visual field coverage. In macaques, the eCaS fell almost exclusively within upper visual field representations of V1. It is worth noting that a less prominent sulcal fold in the dorsal part of the macaque opercular surface contains approximately mirror-symmetric lower visual field representations (Fig. 5). Though this dorsal branch is almost always excluded from descriptions of the external calcarine, it has been considered as a dorsal ramus, or branch, of the eCaS (or lateral calcarine, according to Connolly (1950); “Materials and methods”). When present, the dorsal branch meets the ventral branch at the foveal confluence and extends to the posterior tip of the operculum, therefore, covering the same range of eccentricity as the ventral branch. This visual field bias was also evident in the cortical surface images with the eCaS elongated parallel to the polar angle map (Fig. 5). In humans, the rCaS covered more of the lower visual field representation (especially the most peripheral representations). In macaques, rCaS covered both the upper and lower visual field. Taken together, these macroanatomical landmarks are predictive of the functional organization of V1—especially eccentricity representations—*within species*, but not *across species*.

## Discussion

“Above all, the striking fact that in Man almost the entire striate area is found buried in a long and deep calcarine fissure wholly, or almost wholly, located on the medial face of the hemisphere, whereas in the lower Primates not less than half of the striate area is spread over the lateral face of the occipital lobe, requires a satisfactory phylogenetical explanation.” (Polyak 1957), Pg. 467.

Consistent with the classic quotation from Polyak (1957) above, modern measurements with functional magnetic resonance imaging (Brewer et al. 2002; Fize et al. 2003; Orban et al. 2004; Pinsk et al. 2005; Wandell et al. 2007; Goense et al. 2007; Wade et al. 2008; Wandell and Smirnakis 2009; Kolster et al. 2010, 2014; Arcaro et al. 2011; Livingstone et al. 2017; Arcaro and Livingstone 2017a, b) confirm that V1 extends further onto the lateral surface in macaques than in humans. Our study moves beyond validating well-known differences in the occipital cortical folding structure between macaques and humans. Instead, it draws attention to similarities in the cortical folding structure between species that are often overlooked due to the visualization of neuroimaging data—for example, while flatmaps are common, they often distort the morphology and orientation of sulci such as the retrocalcarine sulcus (rCaS), which is the main focus of the present study. Specifically, our quantifications show that even though the macroanatomical position and morphology of the rCaS is similar between macaques and humans, the rCaS contains different eccentricity representations between species: the human rCaS comprises representations of the central 2°–5° of visual space, while the macaque rCaS comprises representations of the more peripheral 7°–10° of visual space (Figs. 5, 6 and 7). Additionally, the nearby external calcarine sulcus (eCaS) on the lateral surface in both species has a slight lower field bias in humans, and a clear upper field bias in macaques (Figs. 5 and 7). Notably, despite differences in visual field coverage, the macaque eCaS and human rCaS contain similar average eccentricity representations. Given that cortical folding applies mechanical pressures that affect laminar morphology (Hilgetag and Barbas 2005, 2006; Mortazavi et al. 2017) and may have functional significance (Hilgetag and Barbas 2005), resolving the similarities and differences in correspondences between visual maps and cortical folding across primate species as we have done here for V1 is important for understanding the evolutionary and developmental mechanisms of these circuits. We discuss these results in the context of (1) how the same macroanatomical structure can be coupled with different functional representations between species and (2) phylogenetic and anatomical mechanisms that could account for this difference in sulcal–functional coupling between species.

### The same macroanatomical structure can be coupled with different functional representations between species

Morphologically, our results identify a vertical, or bifurcated, portion of the calcarine sulcus on the medial surface toward the occipital pole in human and non-human primates (Huxley 1861; Flower 1862; Cunningham 1892; Retzius 1896, 1906; Smith 1904a, b). Despite historical contentions regarding nomenclature in respect to this cortical expanse (“Materials and methods”), we refer to this portion of the calcarine sulcus that is oriented vertically as the retrocalcarine sulcus (rCaS), which is both consistent with classic (Smith 1904a, b) and modern (Iaria and Petrides 2007; Iaria et al. 2008; Petrides 2019) neuroanatomical studies and distinct from the laterally adjacent eCaS. Within species, our results show a correspondence between part of V1’s retinotopic map and the rCaS (Figs. 5, 6 and 7; Supplementary Figs. 3 and 5). Between species, our results show that this coupling between cortical folding and retinotopic representations is different between macaques and humans with the rCaS corresponding to more peripheral representations in macaques (Figs. 6 and 7). Together, these results indicate that researchers can predict a range of eccentricity representations by locating the rCaS in individual hemispheres within each species: functionally, the rCaS represents eccentricities 7 (posterior lip of rCaS) to 10° (anterior lip of rCaS) in macaques and 2 (posterior lip of rCaS) to 5° (anterior lip of rCaS) in humans. This latter quantification aligns well with recent work showing that “rungs” of the CaS predict eccentricity bands of V1 (Fig. 2). Specifically, Schira and colleagues (2012) show that annectant gyri of the CaS correspond with particular portions of the eccentricity map in V1 in which the second “rung” corresponds to roughly 5°. This second “rung” is just beyond the anterior portion of the retrocalcarine sulcus, which nicely aligns with our data.

To our knowledge, this is the first study to clearly show that the same macroanatomical structure (rCaS) can be coupled with different retinotopic representations between species (Figs. 5, 6 and 7). This is because the cortical positioning (on the medial surface of the cerebral cortex toward the occipital pole) and morphology (vertical bifurcation) of the rCaS is so similar in both macaques and humans, but the relative position of V1’s retinotopic map is shifted medially in humans. Nevertheless, we also acknowledge that the present findings may or may not extend to other anatomical locations in primary or association cortices, which can be tested in future research. For example, the parieto-occipital and calcarine sulci emerge at similar timepoints in gestation (Chi et al. 1977; Nishikuni and Ribas 2013). A number of different retinotopic and functionally specialized areas are located within the POS in both macaques and humans such as V6/PO (Colby et al. 1988; Galletti et al. 2001; Pitzalis et al. 2006, 2010, 2013; Glasser et al. 2016), area prostriata (Sanides and Vitzthum 1965; Sanides 1969; Glasser et al. 2016; Mikellidou et al. 2017), and a scene-selective region (Epstein 2008; Nasr et al. 2011; Epstein and Baker 2019). Future studies can implement a similar approach as in the present study, but applied to cortical areas within the POS. And while we use the POS as an example, this approach can be applied to any sulcus present in both species in any cortical expanse.

### Potential phylogenetic and anatomical mechanisms explaining how the same macroanatomical structure can be coupled with different functional representations between species

Our findings support an interpretation that nearly all of the V1 map is shifted medially in human occipital cortex relative to macaques (Figs. 3D and 5) and cannot be explained by substantial differences in cortical magnification such as an expanded foveal representation in humans (Fig. 6). This is particularly notable given the relatively rigid constraints guiding thalamocortical projections to primary sensory areas early in development. What might explain this difference in anatomical-functional mapping between species? Historically, Polyak (1957) proposed that the change from an arboreal (macaque) to a terrestrial (human) way of life put an increased demand on vision and resulted in an expansion of higher visual areas through natural selection and a decline and recession of the operculum with increased gyrification and folding (a description consistent with a medial shift of V1 in humans compared to macaques). Disproportionate expansion of association cortices in humans likely provided additional pressure for the medial displacement of V1 in humans. In particular, high-expansion regions concentrated in lateral temporal and lateral parietal cortex (Hill et al. 2010) may have effectively pushed relatively low-expansion regions such as primary visual cortex medially. Notably, the surrounding extrastriate areas V2 and V3 are also shifted medially in humans (Brewer et al. 2002; Arcaro and Kastner 2015). Thus, mutations that were beneficial for increased visual and cognitive capabilities in humans likely increased the number or surface area of higher visual and association areas thereby applying pressure for V1, whose map is well preserved across primates, to shift medially.

It is likely that underlying changes in microarchitecture and connectivity accompany shifts in cortical localization. Co-occurring with V1’s displacement is an increase in myelination on the medial surface of the occipital lobe in humans compared to macaques and chimpanzees (Bryant et al. 2019). Additionally, the foveal portion of V1 shows different long-range connectivity in humans and chimpanzees compared to macaques. Specifically, humans and chimpanzees, but not macaques, show long-range anatomical connectivity among the foveal portions of V1 and anterior, inferior, and lateral portions of temporal cortices when using diffusion MRI and tractography analyses (Bryant et al. 2019). These findings are consistent with tract-tracing studies in macaques showing weak connections between V1 and areas in anterior, inferior, and lateral portions of temporal cortices (Doty 1983; Felleman and Van Essen 1991; Rockland and Van Hoesen 1994; Rockland et al. 1994; Gattass et al. 2005; Markov et al. 2011, 2014). Nevertheless, the fact that chimpanzees still exhibit (1) a prominent lunate sulcus and (2) a laterally displaced V1 suggests that the “rolling back and folding in” (to paraphrase Polyak) of striate cortex was a gradual evolutionary change, which can be further explored in future studies.

Causal support for a relationship among connectivity, cortical folding, and the microarchitecture of V1 extends from classic (Rakic 1988; Dehay et al. 1989, 1996; Rakic et al. 1991) and modern (Magrou et al. 2018) studies examining the effects of enucleation during different stages of development and the connectivity and microarchitecture of V1, as well as the morphology of the calcarine sulcus and the operculum. Specifically, enucleation influences the folding of the operculum, as well as the cytoarchitecture and morphology of the portion of V1 within the rCaS (Dehay et al. 1989, 1996). In terms of cortical folding, 5–8 “new” sulci can appear on the operculum that are not present in typically developing macaques (Dehay et al. 1996). Relatedly, enucleation influences thalamocortical and cortico-cortical connections of the developing macaque brain (Magrou et al. 2018). As morphological and connectivity features (Butt et al. 2013; Bock et al. 2015; Andelin et al. 2019) of V1 are also different between blind and sighted human participants, future studies comparing the morphology of the rCaS between blind and sighted participants, as well as using the rCaS as a seed in anatomical and functional connectivity analyses, would serve as a natural next step building on the present and previous work in both humans and non-human primates.

We further highlight that microarchitecture and connectivity also likely contribute to the consistency in the sulcal–functional mapping identified here. For example, previous measurements show a consistent topological relationship between polar angle representations and transcallosal connections in both monkeys and humans. In monkeys, a number of studies showed that transcallosal fibers terminate along the vertical meridies separating a series of visual areas, the most relevant of which for the present paper is the V1/V2 boundary (Zeki 1977; Van Essen et al. 1982; Burkhalter et al. 1986; Kennedy et al. 1986; Felleman et al. 1997). In humans, studies implementing either dMRI and tractography (Dougherty et al. 2005) or polarized light imaging (Caspers et al. 2015) identified structure–function relationships between topological positions within the splenium of the corpus callosum and topological positions of the CaS. Additionally, the V1/V2 border has distinctive myeloarchitectonic (Sanides and Vitzthum 1965; Caspers et al. 2015) and cytoarchitectonic (von Economo and Koskinas 1925; Amunts et al. 2000) features. Thus, the relationship among polar angle representations, connectivity, and microarchitecture can now be revisited when also considering the rCaS in future studies in either species.

## Conclusion

Here, we examined the structural–functional relationship between the retrocalcarine (rCaS) and external calcarine (eCaS) sulci and retinotopic representations within V1 in macaques and humans using anatomical and functional MRI. We find a consistent sulcal–functional relationship across individuals separately within each species, but a different sulcal–functional relationship among individuals between species. Specifically, the rCaS represents eccentricities 2°–5° in humans, but 7°–10° in macaques. These results indicate that the same macroanatomical structure can be coupled with different functional representations between species. Likely inter-related phylogenetic and anatomical mechanisms contribute to the fact that the same macroanatomical structure can be coupled with different functional representations between species. Future studies examining whether sulcal–functional mappings also diverge in other cortical regions will help us better understand the development and evolution of the cerebral cortex across species.

## Supplementary Information

Below is the link to the electronic supplementary material.

Supplementary file1 (PDF 668 KB)

## Data Availability

Data will be made available from the first author upon request.
