# Supplementary material for: The retrocalcarine sulcus maps different retinotopic representations in macaques and humans
Source: Brain Struct Funct. 2021 Dec 17;227(4):1227–45. doi: 10.1007/s00429-021-02427-0 (PMC9046316; doi:10.1007/s00429-021-02427-0)
Supplement: Supplementary file 1 — Supplementary file1 (PDF 668 KB) [file 429_2021_2427_MOESM1_ESM.pdf]

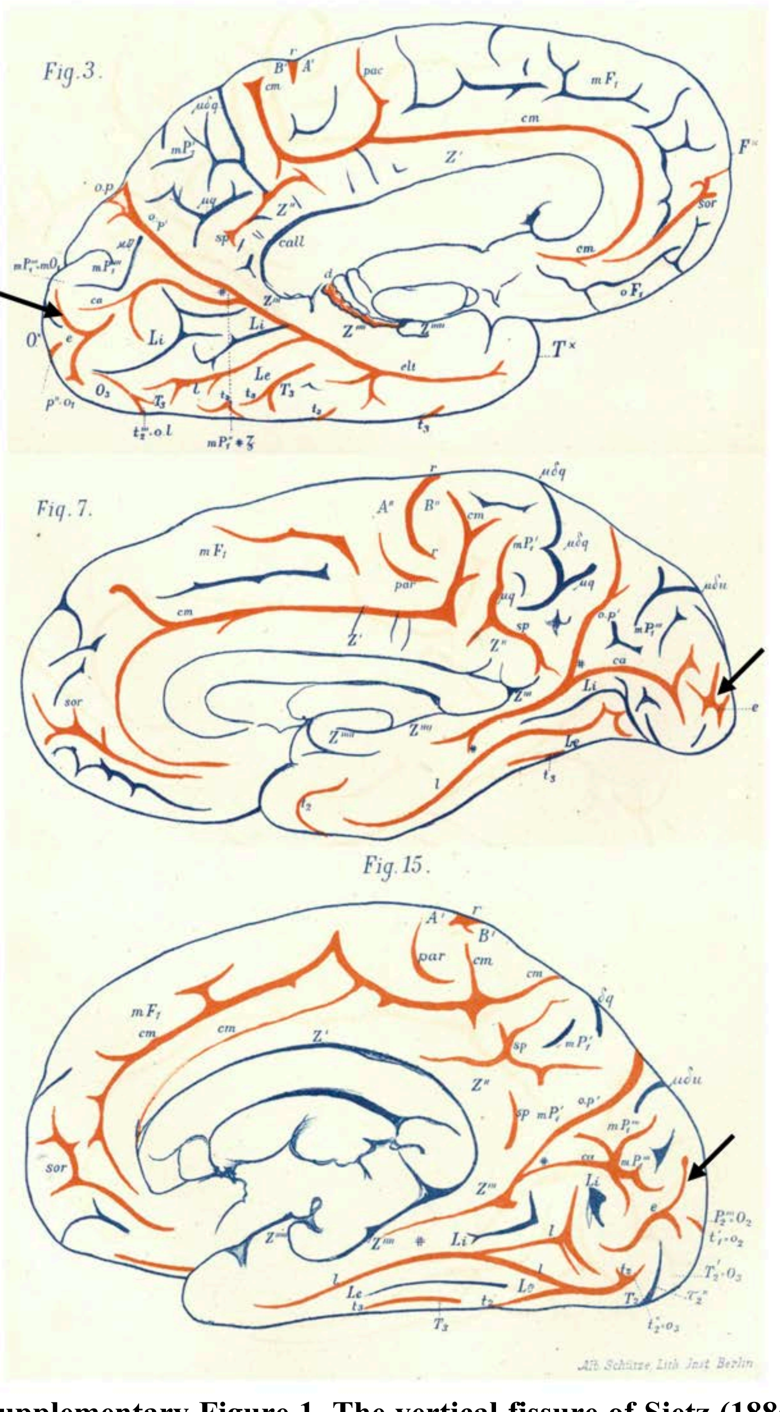

Supplementary Figure 1. The vertical fissure of Sietz (1886). Cunningham (1892) referred to what is now known as the retrocalcarine sulcus (rCaS) eponymically as the vertical fissure of Sietz (1886). In a footnote, Cunningham (1892) writes: "In connexion with this it is interesting to note that in the two…brains described by Sietz, the hinder part of the posterior calcarine fissure is represented as being separate and distinct in three out of the four hemispheres…See his figures 3, 7, and 15" (Cunningham, 1892, pg. 36). Figures 3, 7, and 15 from Sietz (1886) are included here for reference. Black arrows have been added to highlight what Sietz refers to as *e* in the figures and "Endfurche, F. extrema" in the main text.

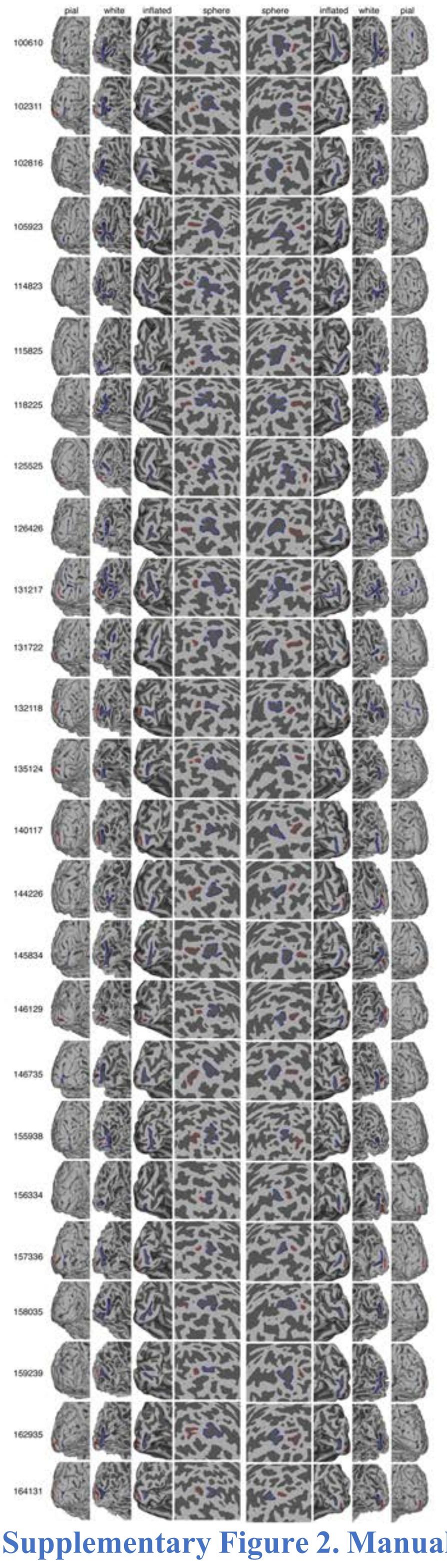

Supplementary Figure 2. Manual definitions of the rCaS and the eCaS in the 48 hemispheres examined in the present study. Medial views of the pial, white, partially inflated, and spherical surfaces from the left and right hemispheres for the 48 human hemispheres that were manually labeled in the present study. 6-digit numbers correspond to HCP IDs. ROIs for the rCaS and eCaS are outlined in red and blue, respectively.

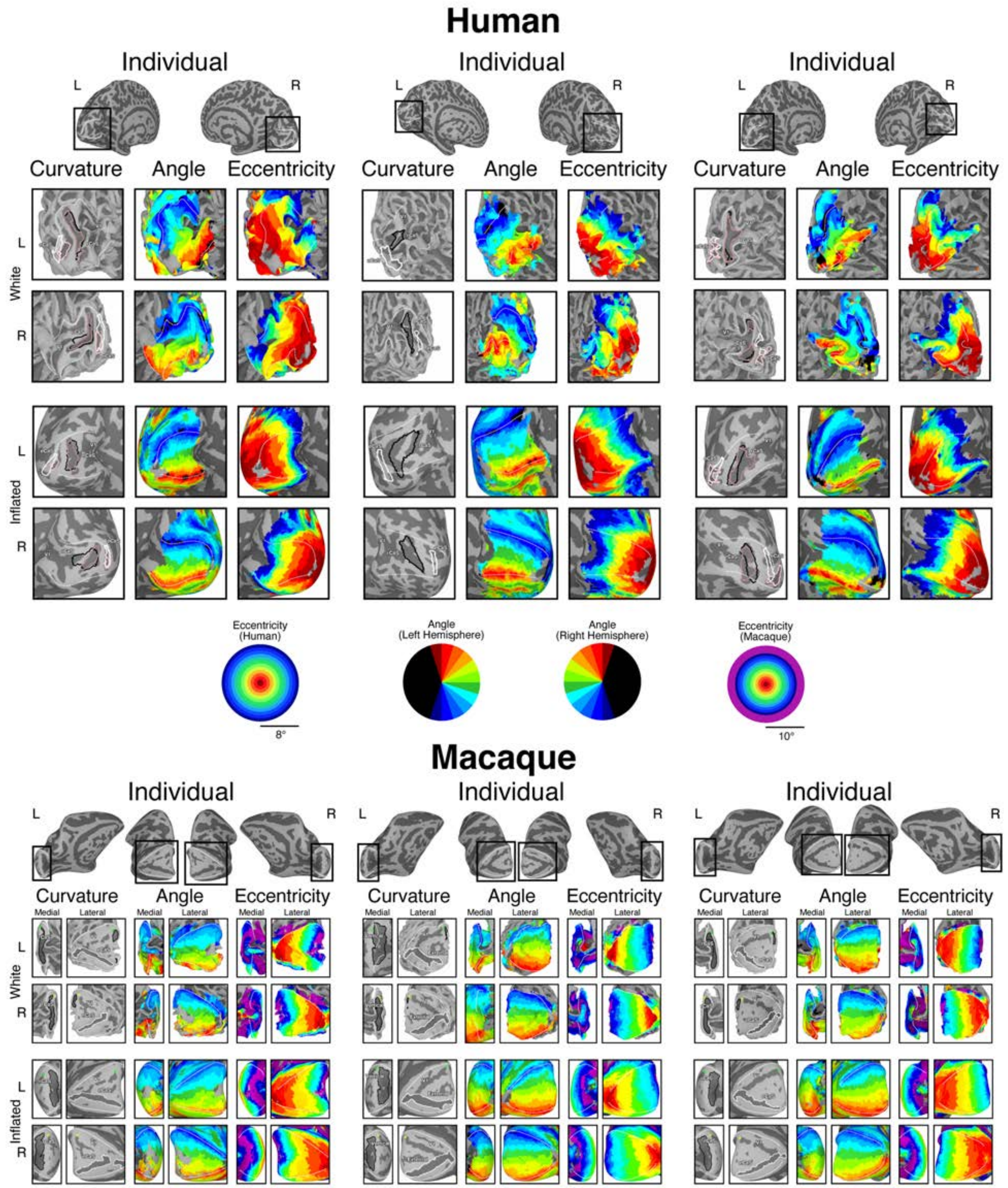

Supplementary Figure 3. Retinotopic correspondence between rCaS and eCaS in individuals. Outlines of the rCaS (black solid line) and eCaS (white solid line) on cortical surface curvature, polar angle, and eccentricity maps in three individual (Top) humans and (Bottom) macaques. Top, left to right: Human participant HCP IDs 157336, 165436, and 162935. Bottom, left to right: Macaque participants M2, M3, and M5. See Figure 5 for additional conventions.

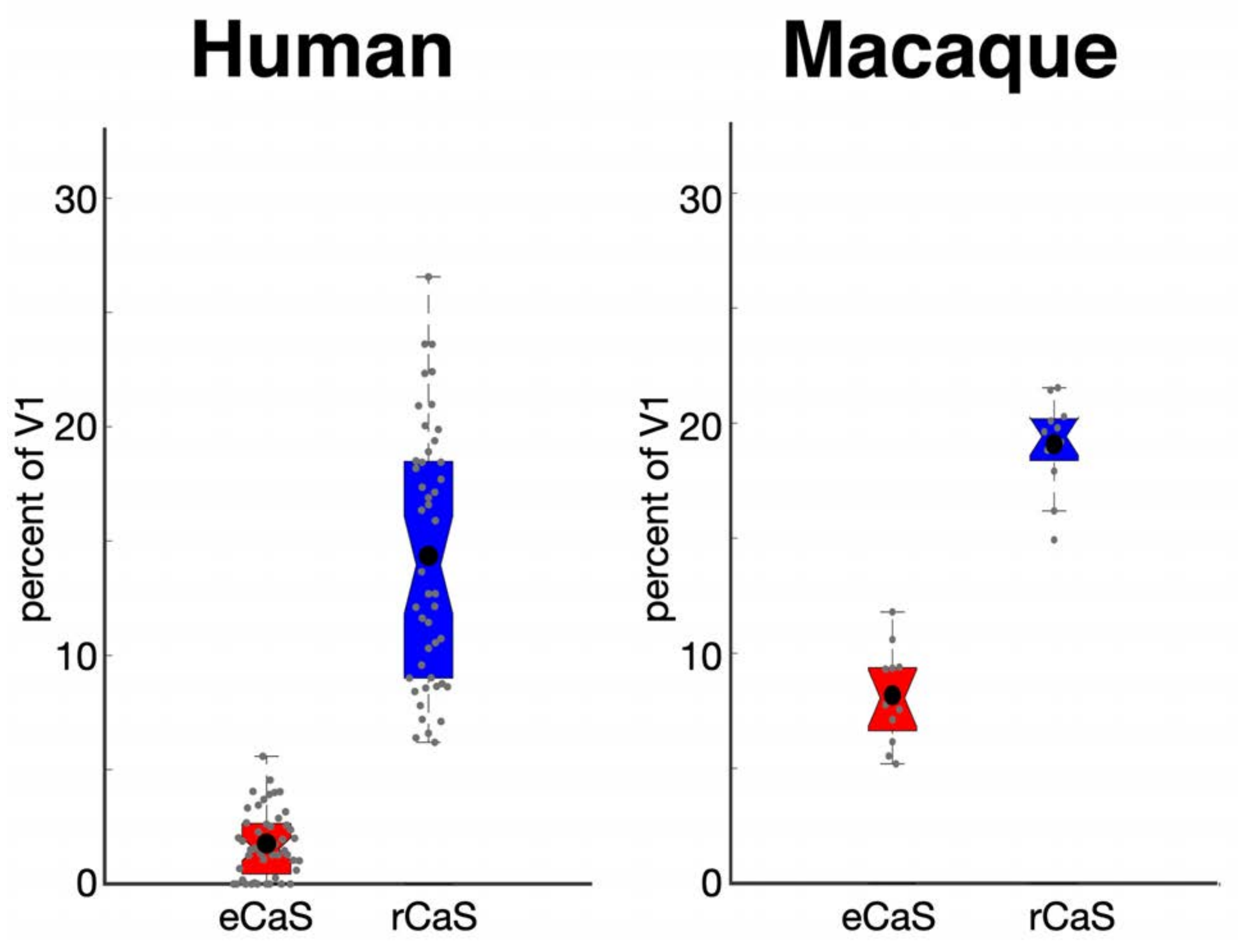

Supplementary Figure 4. rCaS and eCaS surface area relative to V1. Surface area of the rCaS (blue) and eCaS (red) relative to V1 for individuals (grey circles) and group averages (black circle) for (Left) humans and (Right) macaques. Only surface nodes that fell within the extent of V1 for each sulcus were included in this analysis.

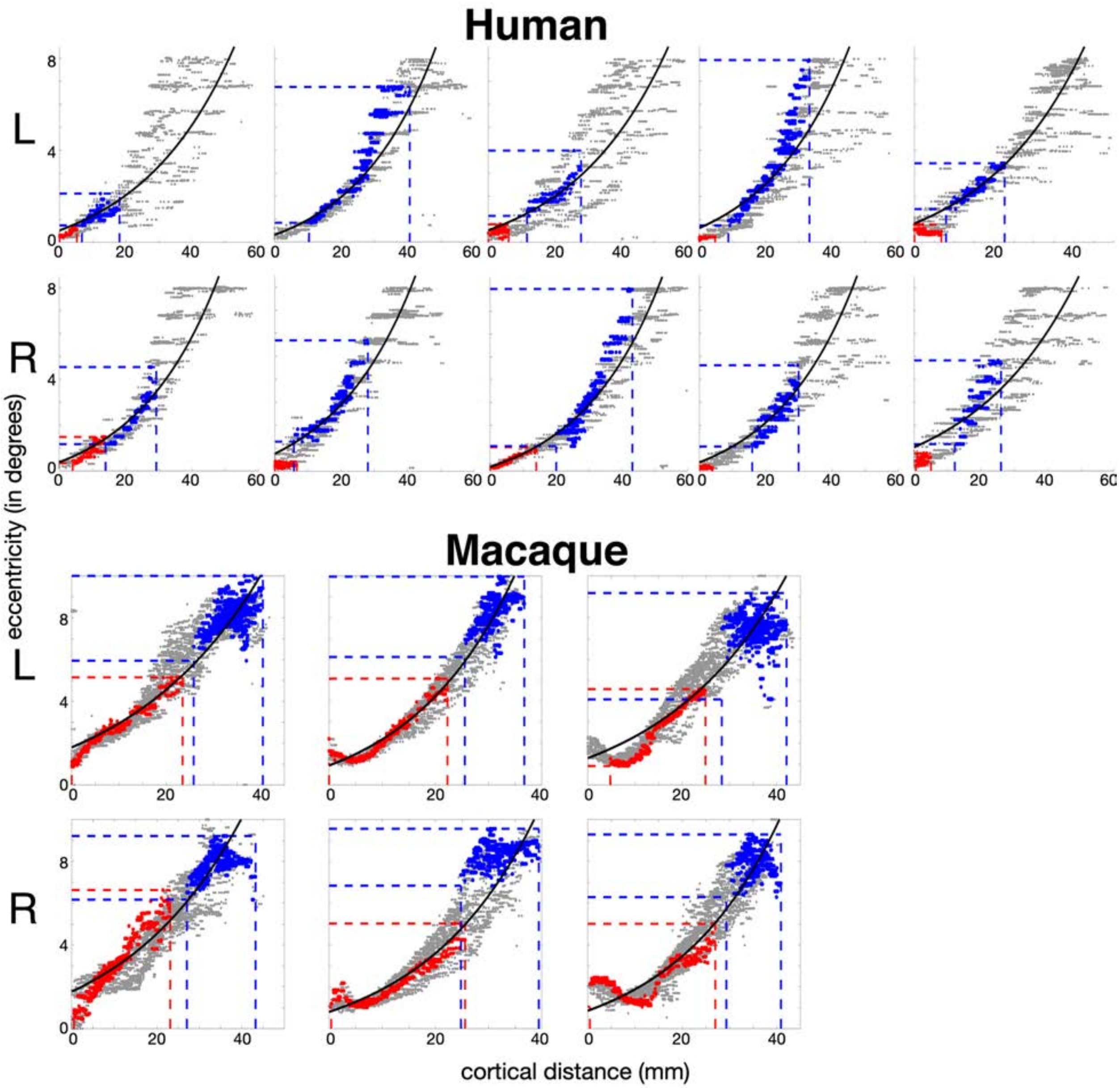

Supplementary Figure 5. Eccentricity representations of rCaS and eCaS as a function of cortical distance from the foveal confluence of V1 in individual participants. Scatter plots of eccentricity representations in relation to cortical distance from the fovea of V1 within the rCaS (blue), the eCaS (red), and the rest of V1 (grey) for (Top) five humans and (Bottom) three macaques. Top, left to right: Human participant HCP IDs 156334, 158035, 159239, 162935, and 164131. Bottom, left to right: Macaque participants M4, M5, and M6. See Figure 6 for additional participants and conventions.
